# Switching from Nusinersen to Risdiplam: A Croatian Real-World Experience on Effectiveness and Safety

**DOI:** 10.3390/jpm14030244

**Published:** 2024-02-24

**Authors:** Andrej Belančić, Tea Strbad, Marta Kučan Štiglić, Dinko Vitezić

**Affiliations:** 1 Department of Clinical Pharmacology, Clinical Hospital Centre Rijeka, 51000 Rijeka, Croatia; dinko.vitezic@uniri.hr; 2 Department of Basic and Clinical Pharmacology with Toxicology, Faculty of Medicine, University of Rijeka, 51000 Rijeka, Croatia; 3 Croatian Health Insurance Fund, 10000 Zagreb, Croatia; tea.strbad@hzzo.hr; 4 Primorje-Groski Kotar County Community Health Centre, 51000 Rijeka, Croatia; marta.kucan@ri.t-com.hr

**Keywords:** effectiveness, nusinersen, real-world data, risdiplam, spinal muscular atrophy

## Abstract

(1) Background: Data on combination or sequential treatment of spinal muscular atrophy (SMA) with disease-modifying drugs (DMDs) are missing and the latter field is poorly understood. The currently available data of patients on risdiplam previously treated with nusinersen are coming from exploratory research mainly focused on safety. Our aim was to investigate the real-world effectiveness (hypothesising non-inferiority) and safety profile of risdiplam in a paediatric-and-adult nusinersen–risdiplam spinal muscular atrophy switch cohort. (2) Methods: A retrospective and anonymous collection of relevant demographic and clinical data for all Croatian SMA patients switched from nusinersen to risdiplam up to September 2023 (reimbursed by Croatian Health Insurance Fund—CHIF) was performed using the CHIF database and associated reimbursement documentation. Patients were included in effectiveness and safety analysis if they met the following inclusion criteria: (i) risdiplam was reimbursed by the CHIF; (ii) the patient received at least six doses of nusinersen before the switch to risdiplam; (iii) there was no relevant pause between the latter disease-modifying drugs; (iv) availability of all prespecified studied data and parameters. (3) Results: In total, 17 patients met the inclusion criteria (58.9% female; median age 12.75 (3.0–44.5) years). In our ‘switch’ cohort, we demonstrated a non-inferiority of risdiplam to nusinersen in the SMA 1 (+1.0 in CHOP INTEND; *p* = 0.067), SMA 3p (+0.7 in HFMSE; *p* = 0.897), and SMA 3a (+0.8 in RHS; *p* = 0.463) subpopulations, during a one-year follow-up period. There were no reports on respiratory function worsening, feeding worsening, and no lethal events. No new safety concerns were identified, except for the weight gain that arose as a new potential adverse drug reaction ‘signal’ in two patients. (4) Conclusions: We have reported pivotal real-world findings on switching SMA patients from nusinersen to risdiplam and demonstrated its effectiveness (non-inferiority), safety, and tolerability in a heterogenous paediatric-and-adult ‘switch’ cohort; this will further increase the quality and standards of care as well as safety of a notable portion of SMA patients, especially for those who demand the switch from nusinersen to other DMDs for clinical or personal reasons.

## 1. Introduction

Gene deletions or mutations affecting the survival motor neuron (SMN1) gene cause spinal muscular atrophy (SMA), a rare autosomal recessive neuromuscular disorder. It is characterised by progressive symmetrical muscle weakness and atrophy due to loss of lower motor neurons in the brainstem and spinal cord [1]. Based on the age at which symptoms first appear, the greatest motor milestone acquired, and the extent of the symptoms, SMA can be divided into five types: 0—prenatal/foetal, 1—non-sitters, 2—sitters, 3, and 4—walkers. Clinical phenotypes widely range from death within weeks of birth to only mild proximal weakness developing during adulthood [2,3]. Growing knowledge on the pathophysiology of SMA alongside breakthroughs in genetic therapeutics have led to the discovery of gene replacement agents, antisense oligonucleotides, and splicing modifiers that improve life span, motor function, and decrease morbidity, even among patients with the most severe clinical forms [2,3,4,5].

Nowadays, after the discovery of new disease-modifying drugs (DMDs) on top of nusinersen (as a pioneer option with the intrathecal route of administration), especially due to the availability of options with peroral and intravenous applications, patients can switch treatments. The switch may be indicated due to an inadequate clinical response or more frequently due to easiness of applications and unwanted side effects from the medicine or its administration. Furthermore, intrathecal nusinersen administration could be challenging or even impossible in patients with reduced spinal access due to severe scoliosis or scoliosis surgery-related spinal fusion [6,7]. Thus, from a safety and effectiveness perspective, collecting more scientific evidence and experience on a switch from nusinersen to other therapies is of high importance [5,8].

Risdiplam is the first and only orally administered SMA DMD; a SMN2-directed splicing modifier, which consequently boosts its effect via producing full-length and functional SMN protein. Due to results pointing toward a favourable benefit to risk ratio accompanied by improvement of motor function in both infantile-onset and late-onset SMA (FIREFISH and SUNFISH clinical trials, respectively), it was approved by the FDA (in 2020) and the EMA (in 2021) and is available on the market for treatment [9,10,11]. Up to now, real-world findings (although scarce) on effectiveness, safety, and tolerability of risdiplam are predominantly congruent with the data obtained from pivotal clinical trials [9,10,12,13].

Our previous research in a real-world setting confirmed the effectiveness and safety of nusinersen in Croatian paediatric and adult SMA patients [14]. Data on combination or sequential treatment of SMA with DMDs are missing and the latter field is poorly understood. The currently available data of patients on risdiplam previously treated with nusinersen are coming from exploratory research mainly focused on safety (the examples can be seen from the JEWELFISH (NCT03032172) study and the Kwon et al. expanded access program report) [15,16]. To the best of our knowledge, our study is a pioneer one aiming to investigate the real-world effectiveness (hypothesising non-inferiority) and safety profile of risdiplam in a paediatric-and-adult nusinersen–risdiplam SMA switch cohort.

## 2. Materials and Methods

The Croatian Health Insurance Fund (CHIF) database and associated reimbursement documentation were used to identify all Croatian SMA patients that were switched from nusinersen to risdiplam (reimbursed by the CHIF) up to September 2023.

The indication for risdiplam introduction (which also corresponds to the CHIF reimbursement criteria) is the diagnosis of 5q SMA 1–3 or one to four copies of SMN2. In order to obtain a reimbursement for risdiplam by the CHIF, the Drug and Therapeutic Committee of the National Referral Centre for SMA needs to submit the formal request accompanied by relevant clinical documentation. The request and reimbursement applies for the period of the next six months, after which the procedure should be repeated.

Under a de-identified personal code, we retrospectively collected the following: age when the nusineresen–risdiplam switch was implemented, gender, SMA type and stage, genotype (SMN1, SMN2, and NAIP copy numbers), dose of risdiplam and number of reimbursement requests, motor function pre-treatment at the time of the switch (baseline) and during the follow-up (1 years’ time), need for respiratory assistance (mechanical ventilation—MV or bi-level positive airway pressure—BiPAP) and feeding support (percutaneous endoscopic gastrostomy—PEG or nasogastric tube—NGT), and adverse drug reactions (ADRs).

Motor function evaluations were performed as per standardised procedures using validated scales: (i) CHOP INTEND (Children’s Hospital of Philadelphia Infant Test of Neuromuscular Disorders) for non-sitting patients and those who were <2 years old; (ii) HFMSE (Hammersmith Functional Motors Scales Expanded) for all sitters, and for walkers younger than 18 years of age (SMA 3p); (iii) RHS (Revised Hammersmith Scale) for all adult walkers (SMA 3a).

The following inclusion criteria were set: (i) risdiplam was reimbursed by the CHIF; (ii) the patient received at least 6 doses of nusinersen before the switch to risdiplam; (iii) there was no relevant pause between the latter DMDs; (iv) the availability of all prespecified studied data and parameters. After applying the latter criteria, eligible patients were included in further clinical–demographic overview, motor effectiveness, and safety (ADR frequency) analyses.

After collection, data was imported from patient-specific forms to Microsoft Excel 2016 (Microsoft Office) and MedCalc v12.1.3 (MedCalc Software bvba, Ostend, Belgium) for statistical analyses. The Kolmogorov–Smirnov test was used to assess the normality of the distribution. Variable values were described by absolute and relative frequencies, measures of central tendency, and measures of spread. In order to present the initial clinical effectiveness of nusinersen, the Wilcoxon matched pairs signed rank test was used to compare the motor function pre-treatment versus at the time of the switch (baseline). Furthermore, the changes over time in terms of motor function (baseline vs. 6 months after switch vs. 12 months after switch) were analysed using the Friedman test for each SMA type separately. All statistical tests were two-tailed and with a 95% CI. The criterion for statistical significance was set at *p* < 0.05.

The study was conducted in accordance with the Declaration of Helsinki. The data was anonymised and already recorded, and because of the retrospective nature and design a waiver of informed consent was applied.

## 3. Results

### 3.1. Baseline Demographic and Clinical Characteristics

**Figure 1:**
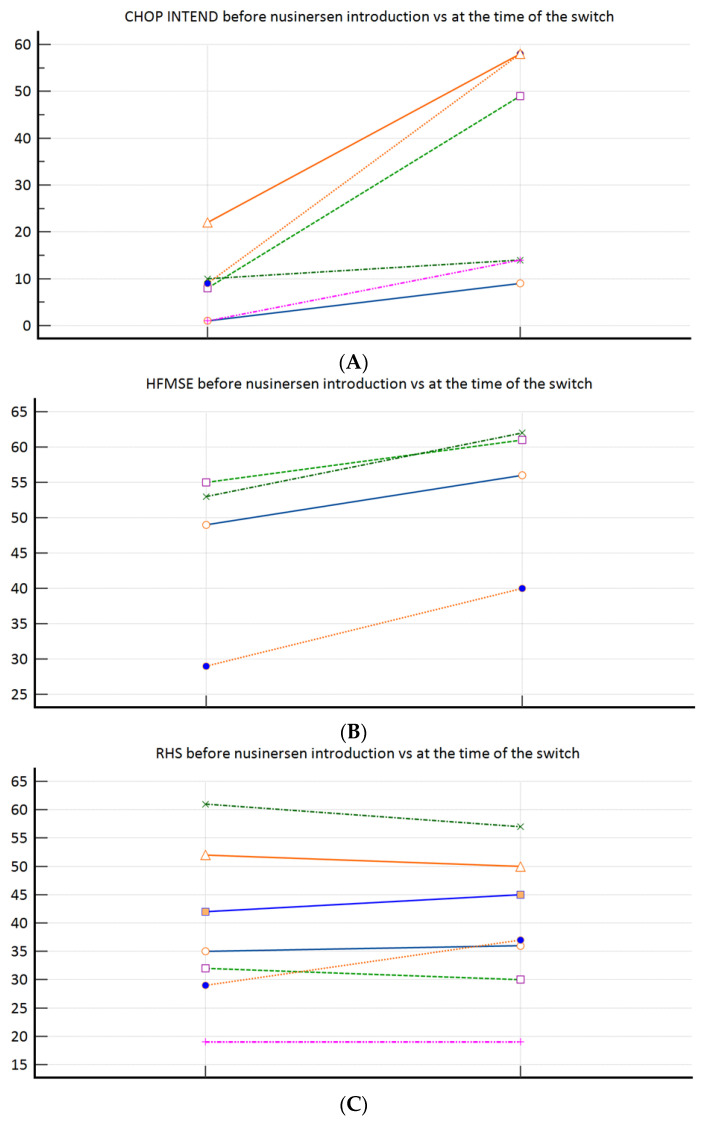
(**A**): CHOP INTEND (n = 6) before nusinersen introduction (8.5 ± 7.7) vs. at the time of the switch (33.7 ± 23.7; *p* = 0.031); (**B**): HFMSE (n = 4) before nusinersen introduction (46.5 ± 11.9) vs. at the time of the switch (54.8 ± 10.2; *p* = 0.125); (**C**): RHS (n = 7) before nusinersen introduction (38.6 ± 14.3) vs. at the time of the switch (39.1 ± 12.7; *p* = 0.916).

During the study period, from 30 SMA patients in total, 17 patients met the inclusion criteria (58.9% female; median age 12.75 (3.0–44.5) years). Baseline (at the time of the nusinersen–risdiplam switch) demographic and clinical characteristics are presented in Table 1. Overall, three patients required MV, and three required BiPAP (all SMA type 1). According to the feeding support data, two patients required PEG and two required NGT (all SMA type 1). Figure 1A–C demonstrates the motor function progress after nusinersen treatment completion (pre-treatment vs. at the time of the nusinersen–risdiplam switch). To elaborate, significant improvement was observed among the SMA 1 patients (Figure 1A), a positive trend was observed among the SMA 3p subpopulation (Figure 1B), whilst there was practically no change when nusinersen was introduced in the SMA 3 patients after the age of 18 years (Figure 1C).

In total, there were 33 positive risdiplam reimbursement requests. The reasons for the nusinersen–risdiplam switch were difficulty performing a lumbar puncture/pain (41.2%; n = 7), post-dural puncture headache (11.8%; n = 2), application preference (parents’ request) (5.9%; n = 1), and not specified (41.2%; n = 7).

### 3.2. Effectiveness Outcomes

**Figure 2:**
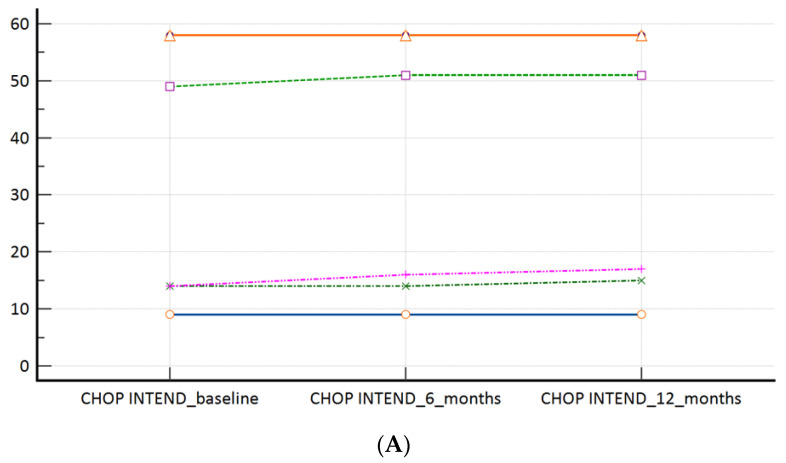

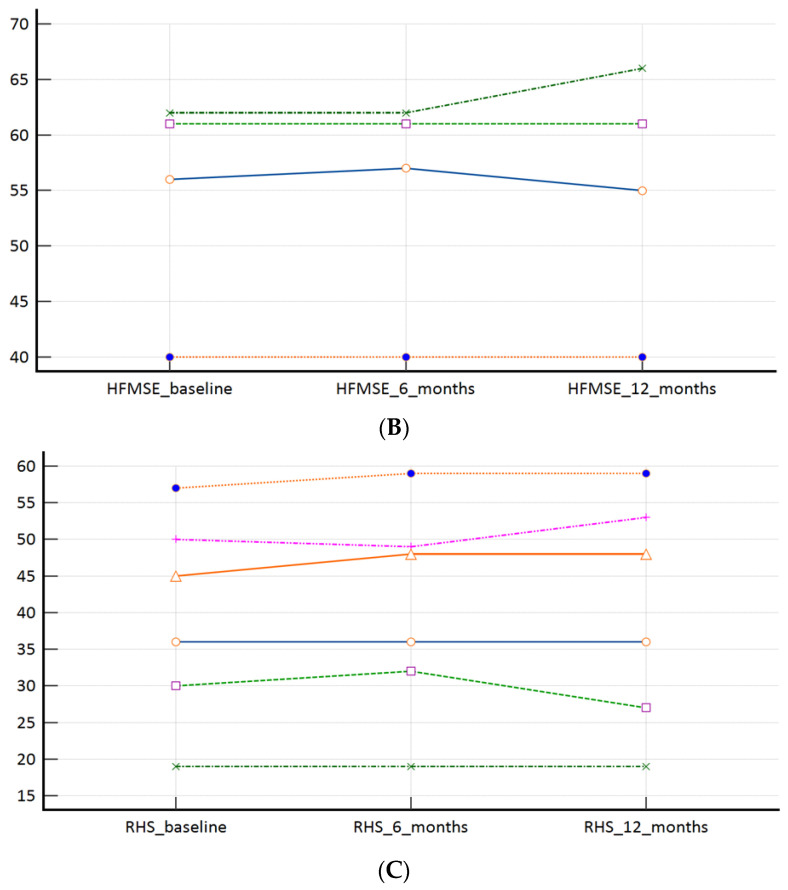
(**A**): CHOP INTEND scores at the time of the switch (baseline) and during the follow-up; (**B**): HFMSE scores at the time of the switch (baseline) and during the follow-up; (**C**): RHS scores at the time of the switch (baseline) and during the follow-up. Measures of central tendency and measures of spread as well as *p* values are presented within Table 2.

In our ‘switch’ cohort, we have demonstrated a non-inferiority of risdiplam to nusinersen, in SMA 1, SMA 3p, and SMA 3a subpopulations, during a one year follow-up period (Table 2, Figure 2A–C). There were no reports on respiratory function worsening (defined as respiratory support introduction or switch from BiPAP to MV), feeding worsening (defined as introduction of PEG or NGT), and there were no lethal events during the study period.

### 3.3. Safety Outcomes

The safety profile was in accordance with the already-known information from the summary of product characteristics. A new potential ADR ‘signal’ which was noticed was weight gain—one SMA 3p patient increased his body weight by 5 kg, while one SMA 3a patient obtained 10 kg after 1 year of risdiplam treatment. At the end of the follow-up, there were two cases of re-switch—they were connected with notable weight gain as well as unsatisfactory motor function dynamics.

## 4. Discussion

A definite forte of our research is the pioneer demonstration of risdiplam’s real-world non-inferiority and safety in SMA patients previously treated with nusinersen. This is important from both safety and effectiveness perspectives, since a portion of patients may experience ADRs or drug administration barriers (e.g., scoliosis/spinal fusion) and may thus demand a new DMD with a peroral (risdiplam) or intravenous (onasemnogene abeparvovec) route of administration.

In the FIREFISH study (patients aged 1–7 months at enrolment, with a genetically confirmed SMA and two SMN2 copies), which was a multicentre, open-label, two-part trial aimed towards demonstrating the safety and efficacy of risdiplam in SMA type 1, 12 infants (29%) were able to sit without support for at least 5 s after 1 year of treatment. Furthermore, 56% of patients within the risdiplam group as compared with 17% among the historical controls achieved a CHOP INTEND score of 40 or higher, whilst 90% as compared with 17% increased at least four points from baseline in the CHOP INTEND score. The results were also promising in terms of survival, event-free survival, as well as in terms of respiratory and feeding domains [9].

In the SUNFISH trial (phase 3, randomised, double-blind, placebo-controlled study, enrolling patients aged 2–25 years with confirmed 5q autosomal recessive SMA type 2 or 3), the primary motor endpoint, defined as a change from baseline in the 32-item Motor Function Measure total score at month 12, was met in a significantly greater number of patients receiving risdiplam compared to the placebo. The risdiplam group also reached statistical significance for secondary objectives such as in the RULM, HFMSE, and SMA Independence Scale scores. Adverse events that were reported in at least 5% more patients who received risdiplam than those who received placebo were pyrexia, diarrhoea, mouth and aphthous ulcers, urinary tract infection, and arthralgias. No significant difference in terms of the rate of serious adverse events was observed [10]. After 24 months, the safety profile remained the same as it had been after 12 months. Also, the benefits of longer-term treatment were confirmed when risdiplam was administered over 24 months, resulting in additional improvement or stabilisation in motor function [12].

The patient/user perspective is always an important additional factor when assessing medicines in general. A recent case series by Powell et al. reported on patient perceptions after switching from nusinersen to risdiplam and concluded that most patients are satisfied when switching those agents, with the method of delivery being a primary factor [17].

As we are waiting for the JEWELFISH study to be finished (which is a multicenter, exploratory, non-comparative, open-label study evaluating safety, tolerability, and the PK/PD relationship of risdiplam in paediatric and adult patients with SMA who are 6 months to 60 years of age at screening, and who have been previously treated with other approved SMA DMDs [15]), our pivotal reports are of great importance.

We have again demonstrated nusinersen’s effectiveness (motor function improvement) in SMA 1 and SMA 3a, whilst its effectiveness was again questionable in the SMA 3p subpopulation, where we can talk about motor function preservation only (Figure 1A–C). Those observations confirmed our previous conclusions provided from our bigger real-word nusinersen cohort, that we recently published in the *Journal of Clinical Medicine* [14]. Our study clearly demonstrated risdiplam as a non-inferior treatment option, when compared to nusinersen in SMA 1, SMA 3p, and SMA 3a subpopulations (Table 2). When a more in-depth look is given to the SMA 1 subpopulation results (Table 2, Figure 1A), there is a potential positive trend in favour of risdiplam for motor effectiveness (e.g., since the p value is very close to significance on a relatively small sample). The latter is in concordance with the data and conclusions provided by Ribero et al., who positioned risdiplam as a superior option, compared to nusinersen, solely for SMA type 1 (and probably non-inferior for other types) [18]. A potential explanation is the fact that risdiplam, on the contrary to nusinersen, increases functional SMN protein levels in both the CNS and peripheral tissues; thus, not solely targeting the motor neurons of the spinal cord [19]. Also, as per the exploratory observations from risdiplam’s RCTs, motor function generally seems to improve more in younger individuals, whilst in older individuals it is predominantly stabilised while on DMDs, possibly due to the irreversibility of motor neuron deterioration.

By presenting this data, we are once again supporting SMA orphan drugs’ efficacy and safety on a wider, heterogenous sample (compared to RCT models in rare diseases), as well as gaining and providing further insights with DMT individualisation, switch, and comparisons [14]. Besides being important for regulatory issues (pricing, reimbursement, and potential shift of available financial resources), by providing experience and concrete evidence it also upgrades SMA patients’ clinical care, quality of life, and options for management if/when the ineffectiveness or ADRs of primary introduced DMT arise.

On top what is already known and mentioned in risdiplam’s summary of product characteristics, we here report on a new potential pharmacovigilance ‘signal’, which is weight gain that arose in one patient with SMA 3p (5 kg) and one with SMA 3a (10 kg). To the best of our knowledge, no evidence on this was reported within in-human trials; only an animal study by Porier et al. reported on increased body weight in mice models [19]. We have sent a formal request to check for potential pharmacovigilance reports on the latter, but no such confirmed cases were identified within the Agency for Medicinal Products and Medicinal Devices (HALMED) and the Eudravigilance database nor through searching periodic safety update reports. Thus, one may find our pivotal observations interesting, and some focus on weight monitoring (or even body composition monitoring by bioelectrical impedance) during risdiplam treatment may be put both for clinicians and scientists for further research.

Although the importance of this study, which presents pivotal findings in real-world effectiveness (non-inferiority) and safety data of risdiplam in a paediatric-and-adult nusinersen–risdiplam SMA switch cohort over a one year period, is indisputable, it is not without limitations. Since this was a retrospective observational study using already collected and aggregated data within the CHIF database and standardised reimbursement documentation, the reported results should be reported accordingly. Unfortunately, due to the high prevalence of scoliosis and spinal fusion among the SMA 2 patients (as it can also be seen from our previous report) they were only occasionally candidates for nusinersen and/or started risdiplam through compassionate use very early, so they did not meet our prespecified inclusion criteria from several reasons; thus, we here only provide scientific evidence for SMA 1, SMA 3p, and SMA 3a [14]. However, since we have proven non-inferiority in SMA 1 and SMA 3, bearing in mind the clinical and pathophysiological characteristics, we are confident that our safety and effectiveness conclusions can be easily extrapolated to the SMA 2 subpopulation also. Among the limitations is definitely the fact that some adverse events that potentially arose during the study period were not included in the medical records, which depends on the leading physician’s/assessor’s quality of evaluation of ADR probability and documentation management habits. However, all patients were strictly followed in the same medical centre (the National Referral Centre for SMA). Standard motor scales were used for treatment effectiveness estimation, which was at all times estimated by starting/initial well-trained assessor to obtain uniformity. Besides the novelty of the findings, another advantage is the presentation of detailed genotype data for the entire cohort as well as conduction of individual motor effectiveness subanalyses for subpopulations such as SMA 3p and SMA 3a.

## 5. Conclusions

There is an obvious clinical (effectiveness and safety) and regulatory (e.g., reimbursement and pricing decisions, and shift of financial resources for indications with proven benefit) need for continuous regional and local data collection by national health insurance providers, at the national healthcare level as well as through disease registry networks, especially in the setting of rare diseases where expensive orphan therapeutic options are available. Hence, we have reported pivotal real-world findings on switching SMA patients from nusinersen to risdiplam and demonstrated its effectiveness (non-inferiority), safety, and tolerability in a heterogenous paediatric-and-adult ‘switch’ cohort; this will further increase the quality and standards of care as well as safety of a notable portion of SMA patients, especially for those who demand the switch from nusinersen to other DMDs for clinical or personal reasons.

## Figures and Tables

**Table 1:** Overview of baseline (at the time of the switch from nusinersen to risdiplam) demographic and clinical parameters of all patients, including sub-groups according to SMA types.

Baseline Demographics		SMA 1		SMA 3p		SMA 3a		SMA 3 Total	Total
**N (% out of total)**		6 (35.3%)		4 (23.5%)		7 (41.2%)		11 (64.7%)	17 (100%)
**Age (years)**	X ± SD	8.0 ± 3.6		11.4 ± 4.3		34.5 ± 8.1		26.1 ± 13.4	19.7 ± 14.0
	Med (Min–Max)	8.0 (3.0–12.75)		10.1 (7.75–17.5)		35.0 (21.25–44.5)		28.5 (7.75–44.5)	12.75 (3.0–44.5)
**Male/female**		3/3		0/4		4/3		4/7	7/10
**SMN1 exon 7 copy number**	0	6		4		7		11	17
	1	0		0		0		0	0
**SMN1 exon 8 copy number**	0	6		3		4		7	13
	1	0		1		2		3	3
	2	0		0		1		1	1
**SMN2 exon 7 copy number**	1	0		0		0		0	0
	2	5		1		0		1	6
	3	1		3		4		7	8
	4	0		0		3		3	3
**SMN2 exon 8 copy number**	1	0		0		0		0	0
	2	5		0		2		2	7
	3	1		3		4		7	8
	4	0		1		1		2	2
**NAIP copy number**	0	4		1		0		1	5
	1	1		0		2		2	3
	2	1		3		4		7	8
	3	0		0		1		1	1
**Mechanical ventilation**—N (% out of total for corresponding SMA type)		3 (50.0%)		0 (0%)		0 (0%)		0 (0%)	3 (17.6%)
**BiPAP**—N (% out of total for corresponding SMA type)		3 (50.0%)		0 (0%)		0 (0%)		0 (0%)	3 (17.6%)
**PEG**—N (% out of total for corresponding SMA type)		2 (33.3%)		0 (0%)		0 (0%)		0 (0%)	2 (11.8%)
**NG tube**—N (% out of total for corresponding SMA type)		2 (33.3%)		0 (0%)		0 (0%)		0 (0%)	2 (11.8%)
**Baseline motor function** (N of patients)	X ± SD	CHOP INTEND	33.7 ± 23.7	HFMSE	54.8 ± 10.2	RHS	39.1 ± 12.7	N/A	N/A
	Med (Min–Max)		31.5 (9–58)		58.5 (40–62)		37 (19–57)		

Note: SMA—spinal muscular atrophy; a—adult; p—paediatric; MV—mechanical ventilation; BiPAP—bi-level positive airway pressure; PEG—percutaneous endoscopic gastrostomy; NG—nasogastric; CHOP INTEND—Children’s Hospital of Philadelphia Infant Test of Neuromuscular Disorders; HFMSE—Hammersmith Functional Motor Scales Expanded; RHS—Revised Hammersmith Scale.

**Table 2:** The average motoric scores (CHOP INTEND, HFMSE, and RHS, respectively) by the time points in SMA 1, SMA 3p, and SMA 3a patients.

Time of Evaluation	N of Patients		Motoric Score/X ± SD		Change from Baseline		*p* Value	
SMA 1								
Baseline	6		33.7 ± 23.7		N/A		0.067	
6 months after switch	6		34.3 ± 23.6		0.6			
12 months after switch	6		34.7 ± 23.3		1.0			
**SMA 3p**								
Baseline	4		54.8 ± 10.2		N/A		0.897	
6 months after switch	4		55.0 ± 10.2		0.2			
12 months after switch	4		55.5 ± 11.3		0.7			
**SMA 3a**								
Baseline	7 *	6	39.1 ± 12.7 *	39.5±13.9	N/A *	N/A	N/A *	0.463
6 months after switch			40.0 ± 13.1 *	40.5±14.3	0.9 *	1.0	0.141 *	
12 months after switch			N/A *	40.3 ± 15.6	N/A *	0.8	N/A *	

* For all comparisons, the Friedman test was used, except for those marked with *, where the Wilcoxon test was used.

## Data Availability

The datasets generated during and/or analysed during the current study are available from the corresponding author on reasonable request.
